# Allium Stents: A Novel Solution for the Management of Upper and Lower Urinary Tract Strictures

**DOI:** 10.5041/RMMJ.10313

**Published:** 2017-10-16

**Authors:** Zaher Bahouth, Boaz Moskovitz, Sarel Halachmi, Ofer Nativ

**Affiliations:** 1 Department of Urology, Bnai-Zion Medical Center, Haifa, Israel; 2 The Ruth & Bruce Rappaport Faculty of Medicine, Technion–Israel Institute of Technology, Haifa, Israel

**Keywords:** Allium Medical, bladder neck contracture, ureteral stricture, urethral stent, urethral stricture

## Abstract

Stents are widely use in endoscopic urological procedures. One of the most important indications is the treatment of urinary tract strictures. Allium™ Medical has introduced several types of stents for the treatment of different types of urinary tract strictures, based on anatomic location. All the stents are made of nitinol and coated with a co-polymer that reduces encrustations. These stents are self-expandable and have a large caliber and a high radial force. They have different shapes, designed especially for the treatment of each type of stricture. One of the most important features of Allium-manufactured stents is the ease of removal, due to their special unraveling feature. The company has introduced the Bulbar Urethral Stent (BUS) for treatment of bulbar urethral strictures; a rounded stent available in different lengths. Initial data on 64 patients with bulbar urethral stricture treated with the BUS showed a significant improvement in symptoms, with minimal complications and few adverse events. For treatment of prostate obstruction in patients unfit for surgery or unwilling to undergo a classical prostatic surgery, the Triangular Prostatic Stent (TPS) was introduced, which has a triangular shape that fits in the prostatic urethra. Its body has a high radial force attached to an anchor (which prevents migration) through a trans-sphincteric wire (which reduces incontinence rate). Initial data on 51 patients showed significant improvement in symptoms and in urinary peak flow rate, with a relatively small number of complications. The Round Posterior Stent (RPS) was designed for treatment of post radical prostatectomy bladder neck contracture. This short, round stent has an anchor, which is placed in the bladder neck. This stent being relatively new, the clinical data are still limited. Ureteral strictures can be treated with the Ureteral Stent (URS), which is round-shaped, available in different lengths, and has an anchor option (for very distal or very proximal strictures). We have previously published data on 107 URSs inserted in patients with ureteral stricture due to several etiologies, including patients who failed previous treatment. All patients were asymptomatic for a long period of follow-up after stent removal, with only one case of re-stenosis. In this paper, we review the urological “covered” stents produced by Allium Medical with the relevant clinical data available at the present time.

## INTRODUCTION

The use of stents in urology was introduced several decades ago and has increased year by year.^1^ There are several adverse events associated with stents, most importantly: irritative symptoms, migration, infection, stenosis, encrustations, and forgotten stents.^2^ In order to overcome most of these complications, Allium™ Medical (Caesarea, Israel) has introduced several new stents; each is intended for the treatment of a different type of stricture along the urinary tract. The company has also introduced several stents for non-urological use (e.g. cardiac and biliary).

All Allium stents used in urology are intended for temporary use, with the concept of rebuilding a normal epithelium around the stent which should be patent long after the stent is removed. This means that Allium stents are intended, in most cases, to cure the disease. All the stents are made of the same material (nitinol) and coated with the same co-polymer that reduces the rate of encrustation, but each stent has a special design. In order to overcome migration, several of the Allium stents are connected to an anchor which significantly reduces the risk of migration. To overcome the forgotten-stent problem, the company has introduced a novel removal feature, unraveling, which significantly eases the removal procedure, even after a long indwelling time.

All urological stents manufactured by Allium Medical are approved for use in Israel.

In this paper, we review the Allium “covered” stents used in urology, together with the relevant clinical data currently available.

## ALLIUM BULBAR URETHRAL STENT

The Allium Bulbar Urethral Stent (BUS) is a self-expandable, large-caliber, round, metal urethral stent designed for urethral strictures treatment. The stent is constructed of a coiled, super-elastic metal alloy (nitinol) and coated with a co-polymer which prevents mucosal hyperplasia and encrustations.

**Figure 1:**
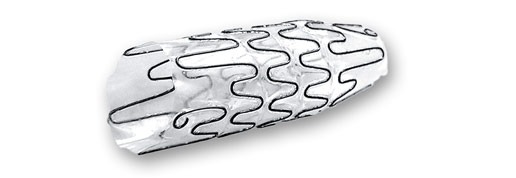
Allium Bulbar Urethral Stent (BUS) The BUS is used for the treatment of bulbar urethral strictures. Picture courtesy of Allium Medical.

The BUS (Figure 1) consists of a proximal soft end attached to a hardened body, which has a large caliber, provides a wide lumen, and keeps urethral patency. The insertion procedure is simple and easy to perform endoscopically, under local or general anesthesia. When inserted, the body—which provides a high radial force—should face the stenotic segment of the urethra. The soft proximal segment prevents sphincteric dysfunction that may cause incontinence.

The stent comes mounted on a 22 Fr specially designed delivery tool; after delivery the stent expands to 45 Fr caliber. The BUS comes in three different lengths: 50 mm, 60 mm, and 80 mm. Moreover, there is an 80-mm reverse-mounted stent designed for use in rare cases when the stricture is in proximity to the urethral sphincter or to the tip of the distal urethra.

The BUS is designed to be used for 12–14 months, after which it should be removed and the urethra would hopefully stay open.

**Figure 2:**
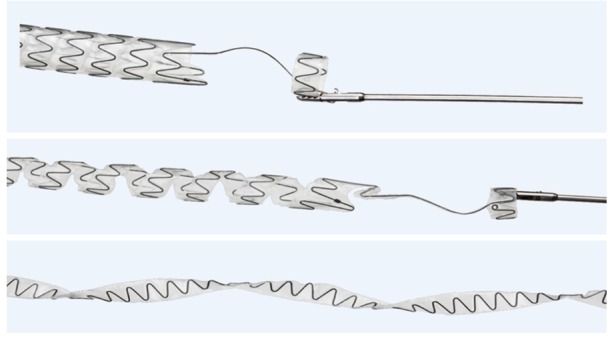
The Allium Unique Unraveling Feature Picture courtesy of Allium Medical.

One of the main advantages of the Allium BUS is its ease of removal: it has a special unraveling feature that turns the stent into a thread-like strip, allowing a non-traumatic, easy, and fast removal (Figure 2).

Insertion and removal can be performed under local anesthesia or general anesthesia according to anesthesiologist and patient preference.

The BUS should not be used in the following situations: age <18 years, acute urinary infection, a contractile bladder, cystolithiasis, or urological implants.

### Clinical Data

We have recently published data on 64 patients who underwent BUS insertion for recurrent bulbar urethral strictures. The median age was 45 years, and all patients had at least one internal urethral dilation surgery. The etiologies of the vast majority of patients were iatrogenic and traumatic. The median stricture length was 20 mm (mean 22 mm, range 10–52 mm). One patient had a long stricture, and we inserted two consecutive urethral stents.

We reported a significant increase in urinary peak flow and significant decrease in International Prostate Symptom score (IPSS); both improved immediately and were also maintained in the long-term follow-up.^3^

In a mean follow-up of more than 2 years, we have shown a patency rate of approximately 75%. At the time of writing, no high-grade complications had been reported. Sixteen patients (25%) had undergone an early stent removal because of: migration (6 patients), progressive deterioration of urinary peak flow (6 patients), and chronic urinary tract infection (UTI) (4 patients).^3^

Because the data are still limited, we did not use the BUS in every indication. Further data should be reported before considering widening indications.

## ALLIUM TRIANGULAR PROSTATIC STENT

**Figure 3:**
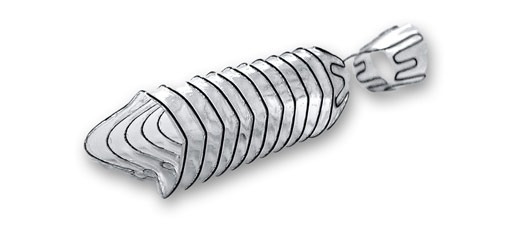
Allium Triangular Prostatic Stent (TPS) The TPS is used for the treatment of prostatic obstruction. Picture courtesy of Allium Medical.

The Allium Triangular Prostatic Stent (TPS) (Figure 3) is designed for the treatment of prostatic obstruction due to benign prostatic enlargement, or prostatic obstruction following thermal or irradiation treatment. Its main use is in patients who are unfit for or unwilling to undergo prostatic surgery.

The stent is highly flexible and, similar to the BUS, is fully covered by a co-polymer, which prevents tissue in-growth and reduces encrustations and calcifications.

As the name suggests, it has a triangular shape that occupies the prostatic urethra in a more natural way compared to circular stents and thus allows for less tissue to be pushed aside and fewer irritative symptoms.

Attached to the main body of the stent—which has a high radial force—is a trans-sphincteric wire, intended to prevent sphincteric dysfunction and decrease the incidence of urinary incontinence. Attached to the wire is the anchor segment, which rests in the urinary bladder and prevents migration.

The TPS is inserted while mounted on a 24 Fr Coude-tip delivery system with radio-opaque markers. After insertion, the TPS opens to a 45 Fr caliber. This stent comes in the following lengths: 30 mm, 40 mm, 50 mm, and 60 mm. Stent removal is as easy as for the BUS.

### Clinical Data

We recently published our experience on 51 patients with benign prostatic obstruction who refused to have classical prostatic surgery or were unfit for surgery for medical reasons. Mean patient age was 72.8 years, the vast majority of them having an American Society of Anesthesiologists (ASA) score of 3 or more. Mean prostatic volume was 38 mL. All patients had severe obstructive symptoms, with an IPSS of 22 or more and maximal urinary flow of 3.3–8 mL/s.^4^

The TPS was inserted successfully in all patients, with no intra-operative complications. All patients had a follow-up of at least 12 months, during which a significant improvement in the obstructive symptoms was noted, with post-stent IPSS of 7.7 and mean peak urinary flow of 16 mL/s.

The TPS was well tolerated, with only 9 patients having urethral pain which resolved for all of them in less than a week; 5 cases had a UTI which resolved except in 1 patient who had a chronic UTI and had the stent removed early. One patient suffered from recurrent gross hematuria that required early removal of the stent. No stent migration or occlusion was reported.^4^

## ALLIUM ROUND POSTERIOR STENT

**Figure 4:**
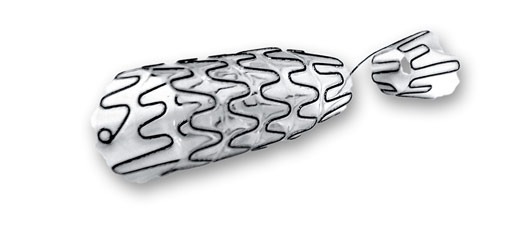
Allium Round Posterior Stent (RPS) The RPS is used for treatment of bladder neck contracture. Picture courtesy of Allium Medical.

The Allium Round Posterior Stent (RPS) (Figure 4) is designed for the treatment of bladder neck or posterior urethral contracture, mainly following prostatic surgery (radical prostatectomy, open prostatectomy, and endoscopic prostatectomy).

The RPS is very similar to the TPS, made of similar material, has a connecting trans-sphincteric wire, and has the same high radial force and large caliber (45 Fr). It differs from the TPS in its round shape (compared to the triangular shape) and in its length (30 mm or 40 mm). The removal procedure is easy thanks to its unraveling feature.

### Clinical Data

The RPS is intended for temporary use (up to 12 months). Experience with the RPS is still limited, and publications are limited to case reports and presentations at international meetings.^5^,^6^

One presentation reporting on the initial experience with the RPS included 10 patients; most of them were incontinent and used a Cunningham clamp for urinary control. Migration was recorded in two patients, and their stents were replaced. In a short follow-up time of 10 months, all stents were patent.^6^

## ALLIUM URETERAL STENT

**Figure 5:**
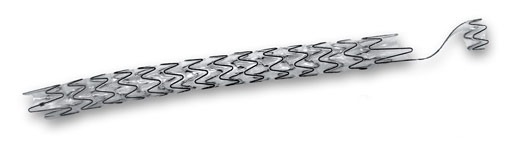
The Allium Ureteral Stent (URS) The URS is used for the treatment of ureteral strictures and includes an anchor (stents intended for use in mid-ureter do not have an anchor). Picture courtesy of Allium Medical.

The Allium Ureteral Stent (URS) (Figure 5) is intended for the treatment of ureteral strictures, caused by any etiology, with a wide range of stricture length. Similar to the previously described stents, it is also made of nitinol and covered with a co-polymer that prevents encrustation and tissue in-growth, has a high radial force to face the stricture, and its insertion can be easily carried out endoscopically with fluoroscopic guidance. It can be inserted either by a retrograde or anterograde approach, usually after balloon ureteral dilation.

The URS comes pre-mounted in an 8 Fr or 10 Fr delivery system, with the following lengths: 60 mm, 80 mm, 100 mm, and 120 mm. It comes in two diameters: 24 Fr and 30 Fr.

A specially designed model of the URS has an anchor segment and can be inserted in two lengths, 100 mm and 120 mm, with a diameter of 24 Fr or 30 Fr. The anchoring part can be placed in the renal pelvis or the bladder, reducing the risk of stent migration. This stent is intended for temporary short- or long-term use.

### Clinical Data

We have previously reported the results of a multi-center worldwide study that our department took part in. This study included 92 patients with 107 stenotic ureters due to different etiologies: gynecological cancer (with or without irradiation), bladder cancer, iatrogenic stricture, uretero-ileal stenosis, stricture following uretero-pelvic junction obstruction repair, and iatrogenic ureteral fistula.^7^

In this study, the stents were planned to be removed after 1 year. However, 21 patients died of their primary disease while carrying the stents. Stent migration was seen in 15 (16%) patients; 4 of them underwent a re-insertion because of early migration; all others had the stents removed without the need for re-stenting. The vast majority of migrated stents were in the mid-ureter, and almost all migrated to the urinary bladder. Some patients were lost to follow-up, and some patients are still in follow-up with indwelling stent.^7^

In a follow-up period of more than 2 years, only one stent was obstructed after 11 months dwelling time. All patients were asymptomatic after stent removal and during the period of carrying the stent.^7^ These stents were successful regardless of the etiology.

The reported adverse effects of the URS are similar to those reported with the use of a pigtail JJ stent, with a somewhat lower incidence of irritative symptoms.^8^

## CONCLUSIONS

Allium Medical has introduced an improvement of a well-known and widely used product in endourological practice—stents. Designed uniquely for each type of stricture, made of nitinol, and coated with a specially designed co-polymer, these stents have advantages over other known stents. Ease of removal is a major advantage and overcomes the problems of forgotten stents. More experience, data, and longer follow-up are needed to better evaluate the short- and long-term usage of these urological stents.

## Abbreviations

ASA: American Society of Anesthesiologists
BUS: Bulbar Urethral Stent
IPSS: International Prostate Symptom Score
RPS: Round Posterior Stent
TPS: Triangular Prostatic Stent
URS: Ureteral Stent
UTI: urinary tract infection.
